# Rapid Epigenetic Adaptation in Animals and Its Role in Invasiveness

**DOI:** 10.1093/icb/icaa023

**Published:** 2020-04-25

**Authors:** Vitor Coutinho Carneiro, Frank Lyko

**Affiliations:** Division of Epigenetics, DKFZ-ZMBH Alliance, German Cancer Research Center, Im Neuenheimer Feld 580, 69120, Heidelberg, Germany

## Abstract

Invasive species represent a serious ecological threat for many ecosystems worldwide and provide a unique opportunity to investigate rapid adaptation and evolution. Genetic variation allows populations of organisms to be both robust and adaptable to different environmental conditions over evolutionary timeframes. In contrast, invasive animals can rapidly adapt to new environments, with minimal genetic diversity. Thus, the extent to which environmental effects can trigger epigenetic responses is particularly interesting for understanding the role of epigenetics in rapid adaptation. In this review, we provide a brief overview of the different epigenetic mechanisms that control gene expression, and emphasize the importance of epigenetics for environmental adaptation. We also discuss recent publications that provide important examples for the role of epigenetic mechanisms in environmental adaptation. Furthermore, we present an overview of the current knowledge about epigenetic modulation as an adaptive strategy for invasive species. A particularly interesting example is provided by the marbled crayfish, a novel, monoclonal freshwater crayfish species that has colonized diverse habitats within a few years. Finally, we address important limitations of current approaches and highlight the potential importance of less well-known mechanisms for non-genetic organismal adaptation.

## Introduction

It is commonly accepted that differences in the deoxyribonucleic acid (DNA) sequence (i.e., genetic variation) provide organisms with the ability to adapt to different environmental conditions. Natural selection acting on genetic variants explains how organisms can colonize ecological niches over evolutionary timeframes. However, several examples of rapid adaptation and invasion are difficult to explain solely by the selection of genetic variants. As such, epigenetic mechanisms have been increasingly used to explain these phenomena. We delimit epigenetic plasticity from transgenerational epigenetic inheritance, which is controversially discussed and would allow the inheritance of acquired epigenetic traits (Heard and Martienssen 2014). In contrast, epigenetic adaptation is effective within single generations.

Our article focuses on epigenetic adaptation in animals, as several reviews are already available for plants (Richards 2011; Pikaard and Mittelsten Scheid 2014). We begin by defining epigenetics, providing a short overview of the different epigenetic mechanisms, and emphasizing the importance of epigenetics for environmental adaptation. We continue with a detailed description of recent studies that provided important paradigms for the role of epigenetic mechanisms in environmental adaptation. This includes the marbled crayfish, a clonally reproducing animal that has recently colonized various ecosystems worldwide. Finally, we identify important open questions and provide suggestions for future development.

## Epigenetic regulation of gene expression

**Fig. 1:**
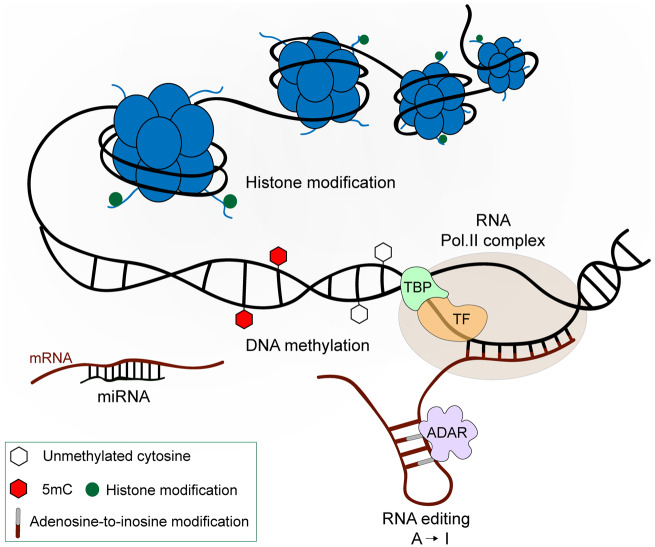
Mechanistic model for epigenetic control of gene expression. Epigenetic mechanisms are important for regulating gene expression and chromatin architecture in eukaryotic cells. The fundamental repeat unit of chromatin is the nucleosome, which is comprised of an octamer of core histone proteins (represented by blue circles). Post-translational modifications (small green circles) of the amino-terminal tails of histone proteins (short blue lines deriving from the histones) affect chromatin structure by fine-tuning the accessibility of the transcription machinery (transcription factors, co-regulators, and the RNA polymerase II complex). DNA methylation (red hexagons) refers to the addition of a methyl group to the five-position of cytosine in the context of CpG dinucleotides. RNA-based mechanisms silence gene expression via complementary base-pairing to mRNA molecules. This evolutionarily conserved mechanism affects gene expression by promoting mRNA degradation or the disruption of protein translation. Adenosine to inosine (A-to-I) RNA editing represents a mechanism to diversify mRNA coding. Because inosine pairs with cytosine, it is a biological mimic for guanosine and can thus alter mRNA coding. The potential of A-to-I editing to diversify the transcriptomic profile represents a possible mechanism to increase phenotypic plasticity and, therefore, aid adaptation to new environments.

Multicellular organisms are genetically homogeneous, but different cell types and functions arise from the differential expression of genes. It has been shown that differentiating mammalian cells undergo dynamic epigenetic changes, resulting in the establishment of cell-type-specific programs (Gifford et al. 2013; Xie et al. 2013; Roadmap Epigenomics Consortium et al. 2015). Transcription factors primarily control gene expression, and nearly all biological processes are linked to post-synthetic modifications of the three fundamental macromolecules: DNA, ribonucleic acid (RNA), and proteins (Fig. 1). These covalent modifications have been commonly termed as “epigenetic” even though this broad definition has often resulted in misconceptions (Deichmann 2016; Henikoff and Greally 2016).

Transcription in eukaryotes takes place in the context of chromatin. In general, nucleosomes impede DNA transcription, either by physically obstructing or by compartmentalizing the binding of transcription factors to DNA. An important feature of histones, and particularly of their N-terminal tails, is their ability to carry post-translational modifications, which can affect chromatin structure in different ways. Many of these modifications are dynamically regulated by families of enzymes that write or erase the modifications (Allis and Jenuwein 2016). Besides their role in chromatin remodeling, histone modifications are also responsible for recruiting effector proteins or disrupting their binding to chromatin.

RNA-based mechanisms have also been involved in epigenetic regulation, but are less well understood in animals. Key molecules are noncoding RNAs that belong to several classes. Small interfering RNAs (siRNAs) and microRNAs are derived from longer precursor RNAs by the action of RNAse III-family enzymes, such as Drosha and Dicer, and can inhibit translation or direct mRNA degradation (Kim et al. 2009; Castel and Martienssen 2013). Additionally, siRNAs can regulate gene transcription through transposable element silencing and the interaction with other epigenetic mechanisms, such as DNA methylation and histone modification (Holoch and Moazed 2015). Finally, it should be noted that additional mechanisms that are not traditionally associated with epigenetic gene regulation have the capacity to increase phenotypic plasticity. A prominent example is provided by mRNA editing (Figure 1). The deamination of adenosine to inosine (recognized as guanosine during translation) by the ADAR family of enzymes has the potential to recode codons and diversify the transcriptome by allowing the translation of alternative protein products from a single gene (Eisenberg and Levanon 2018).

DNA methylation represents the most well-studied epigenetic mechanism, and characterizing the biological relevance of this modification and its impact on gene expression has driven research in the field for a long time (Lyko 2018). The dynamic equilibrium between methylation and demethylation modulates gene expression and can be faithfully propagated through many cell generations (Jones 2012). While often associated with transcriptional silencing in mammals, DNA methylation patterns are complex and can affect gene promoters, gene bodies, and repeats differently (Jones 2012; Schubeler 2015).

Mechanistically, the effects of DNA methylation are often explained by their complex association between DNA methylation and transcription factor binding (Schubeler 2015; Yin et al. 2017). An additional model involves the recruitment of methyl-binding proteins (MBPs), which triggers chromatin structural changes and altered gene expression (Klose and Bird 2006). More specifically, MBPs have been shown to bind to methylated DNA, often at gene promoters, resulting in transcriptional repression through the recruitment of histone deacetylases (Klose and Bird 2006). While these models are well-established in vertebrate systems, the role of DNA methylation in invertebrates remains much less understood. Invertebrate methylation patterns can be highly diverse (Bewick et al. 2017) and are often defined by gene body methylation, while promoter methylation is rarely observed. Gene body methylation may reduce spurious RNA polymerase transcription (Neri et al. 2017), which is also known as transcriptional noise (Faure et al. 2017).

From an ecological perspective, epigenetic mechanisms could promote phenotypic plasticity and adaptation to different environments (Verhoeven et al. 2016). Thus, the extent to which environmental effects can trigger epigenetic responses is particularly interesting for understanding the role of epigenetics in animal adaptation.

## Key examples for rapid epigenetic adaptation

With the emergence of epigenetics and the development of methods for the detection of epigenetic modifications, many studies have attempted to link adaptive changes to epigenetic changes (Feil and Fraga 2012; Hu and Barrett 2017). More recently, however, several key studies have provided more convincing evidence, based on improved study design and more rigorous methodology. In the following, we provide an overview of several of these particularly interesting examples. For instance, it has been shown that DNA methylation patterns of salmon that were reared in artificial hatcheries were different from salmon that were reared in the wild (Le Luyer et al. 2017). While the phenotypic effects of differentially methylated regions were not investigated, the results were based on sound statistical analysis and included controls for genetic polymorphisms, which represent a major confounding factor in DNA methylation analyses. Overall, the study suggested that adaptive epigenetic changes to the hatchery environment underpin the reduced fitness of hatchery-reared salmon when released in the wild.

Similar observations were also made in terrestrial animals. For example, different methylation patterns were identified in the early stages of a founding population of the brown anole lizard (*Anolis sagrei*), suggesting a relationship between epigenetic variation and rapid responses to environmental changes (Hu et al. 2019). It was shown that after a 4-days exposure to a new habitat, the lizards had methylation patterns that were distinct from their original habitat. Interestingly, differentially methylated cytosines were detected at genes with functions likely to be relevant to animal plasticity (e.g., signal transduction, immune response, and circadian rhythm). Further integrative studies using whole-genome bisulfite sequencing and RNA sequencing, should improve the understanding of how DNA methylation modulates phenotypic responses to environmental stressors during the colonization of new habitats.

Epigenetic mechanisms have also been implied in the adaptation of the globally distributed scleractinian coral *Stylophora pistillata* to warmer and more acidic ocean water. Genome-wide DNA methylation analysis revealed pH-dependent differential methylation at genes associated with growth and stress response pathways (Liew et al. 2018). Interestingly, the coral methylome is characterized by gene body methylation, and high levels of gene body methylation were shown to reduce spurious transcription and transcriptional noise (Li et al. 2018). While it appears possible that reduced transcriptional noise facilitates adaptation, variable gene expression has also been suggested as an adaptive mechanism in corals (Kenkel and Matz 2016). Both effects could conceivably be mediated by alterations in gene body methylation.

Another interesting example of an adaptive epigenetic change is provided by certain cavefish, where the environment triggers the degeneration of the eyes. Interestingly, in the Pachón blind morph of the Mexican tetra (*Astyanax mexicanus*), this process is not accompanied by known genetic mutations in eye developmental genes. Instead, it was shown that promoter hypermethylation could repress eye-specific genes and thus results in defective eye development (Gore et al. 2018). Of note, this study also provides promising results from initial functional experiments, such as a partial rescue of eye phenotypes by injection of a DNA methylation inhibitor (Gore et al. 2018).

## Epigenetic adaptation in invasive species

Invasive species represent a serious ecological threat for many ecosystems worldwide and provide a unique opportunity to investigate rapid adaptation and evolution. Invasive animals often show reduced genetic diversity, which is thought to limit the adaptive and evolutionary potential by constraining the availability of new gene variants (Chown et al. 2015). Nevertheless, invasive species are often highly successful in adapting to new and heterogeneous environments, with adaptive plasticity as an essential strategy for rapidly colonizing new habitats and out-competing native species. Commonly neglected or hidden in natural environments, phenotypic plasticity becomes particularly relevant when the invasion of a new or altered environment occurs (Ghalambor et al. 2007; Fox et al. 2019).

Defined as the ability of a genome to express various phenotypes, phenotypic plasticity implies the capacity for multiple adaptive responses to environmental changes, such as environmental stress, rapid growth, and reproduction (Fox et al. 2019). However, the expansion of invasive species represents a genetic paradox, as individuals can adapt rapidly to new, sometimes challenging environments. As discussed before, epigenetic mechanisms can modulate phenotypes without changing genotypes. Through the modulation of gene expression, epigenetic changes can increase phenotypic variation in the absence of genetic diversity, which might facilitate animal adaptation to both biotic and abiotic environmental challenges (Jaenisch and Bird 2003; Duncan et al. 2014).

Epigenetic plasticity can explain organismal adaptation to environmental changes (Hollander et al. 2015). A detailed summary of a significant number of indicative studies that link adaptivity in invasive species to DNA methylation variability has been published recently (Hawes et al. 2018). For example, intrapopulation DNA methylation variability was observed in the invasive house sparrow (*Passer domesticus*) and proposed as a rapid response mechanism to environmental challenges (Liebl et al. 2013; Sheldon et al. 2018). Additionally, in the pygmy mussel (*Xenostrobus secures*), global DNA hypomethylation was detected in a recently evolved population and interpreted as a mechanism to promote phenotypic plasticity and thereby facilitate the expansion of invasive populations (Ardura et al. 2017). Finally, in the invasive whitefly (*Bemisia tabaci*), DNA methyltransferase 1 (DNMT1) knockdown caused reduced thermotolerance, but DNA methylation patterns were not investigated (Dai et al. 2018).

Despite these recent advances, it is worth mentioning that many studies in this field have analyzed DNA methylation using indirect methods and that it will be important to confirm initial findings by more robust and more powerful approaches, such as bisulfite sequencing (Lea et al. 2017). Furthermore, the mechanisms of how the environment shapes the epigenome are mainly unknown. Finally, it will be important to more comprehensively analyze epigenetic gene regulation in invasive animals, in order to better understand and ultimately predict their adaptive and invasive potential. Detailed analyses of select model organisms could be particularly useful for the establishment of more generalizable concepts.

## The marbled crayfish as an example for epigenetic adaptation in an invasive species

The marbled crayfish (*Procambarus virginalis*) represents a novel freshwater crayfish species that emerged from the German aquarium trade about 25 years ago (Scholtz et al. 2003; Lyko 2017). Anthropogenic releases have founded expanding wild populations in several European countries (Chucholl and Pfeiffer 2010; Lipták et al. 2016; Novitsky and Son 2016; Patoka et al. 2016; Pârvulescu et al. 2017; Deidun et al. 2018; Ercoli et al. 2019). Furthermore, marbled crayfish have rapidly invaded ecologically distinct habitats in Madagascar and currently colonize an area that extends over 100.000 km^2^ (Jones et al. 2009; Kawai et al. 2009; Gutekunst et al. 2018; Andriantsoa et al. 2019).

**Fig. 2:**
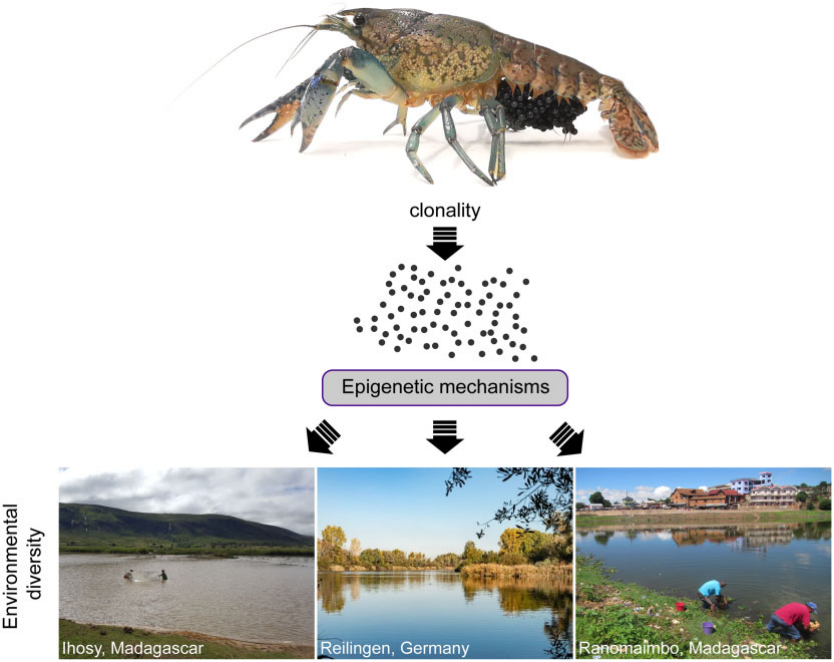
Clonal expansion and rapid adaptation of marbled crayfish. The marbled crayfish is a monoclonal, parthenogenetically reproducing species. Individual animals show considerable phenotypic plasticity and adaptivity to various parameters, such as temperature, osmolarity, and pollution. Epigenetic modulation has been implied to marbled crayfish adaptation, but the precise mechanisms remain to be identified. The top panel picture shows an adult marbled crayfish (3 years old) with eggs. The bottom panel displays three ecologically different locations with established marbled crayfish population: Ihosy River (Madagascar), Lake Reilingen (Germany), and Ranomaimbo lake (Madagascar).

The marbled crayfish is a parthenogenetic descendant of the sexually reproducing slough crayfish (*P. fallax*) from Florida (Martin et al. 2010). Its particular mode of reproduction (obligatory apomictic parthenogenesis) results in the generation of a genetically homogeneous, monoclonal population (Gutekunst et al. 2018). Parthenogenetic reproduction is not uncommon in the animal kingdom, but obligatory parthenogenesis is very rare and has been described as an “evolutionary scandal,” as it runs counter to fundamental tenets of evolutionary biology. As genetic polymorphisms are negligible in the marbled crayfish population, the successful adaptation of the animals to different environments cannot be explained by the Darwinian selection of the genetically best-adapted genotype. It is therefore very likely that marbled crayfish use epigenetic mechanisms, such as DNA methylation, to adapt their genome to specific environmental conditions (Figure 2).

The combination of obligatory apomictic parthenogenesis with an extremely young species age (25–30 years) in marbled crayfish creates unique opportunities for elucidating the role of epigenetic mechanisms in invasiveness. Indeed, the genome of the marbled crayfish encodes a conserved and active DNA methylation toolkit (Gatzmann et al. 2018). A detailed analysis of DNA methylation patterns revealed that that the modification is targeted to the gene bodies of housekeeping genes, similar to many other invertebrates (Gatzmann et al. 2018). Interestingly, gene body methylation was found to be inversely correlated with gene expression variability. When compared to the parent species of marbled crayfish (*P. fallax*), many genes showed reduced levels of gene body methylation and increased levels of gene expression variability (Gatzmann et al. 2018). This might indicate that low levels of gene body methylation promote adaptability (and invasiveness) through increased gene expression variability. This hypothesis will have to be confirmed by a detailed molecular analysis of specific ecotypes and/or by direct experimental approaches that elucidate the functional role of DNA methylation in this organism.

Of note, marbled crayfish possess key prerequisites for a laboratory model, such as suitable size, resistance against handling stress, high fertility, and a relatively short generation time (Vogt 2011). In addition, marbled crayfish combine obligatory apomictic parthenogenesis with an extremely young evolutionary age (25–30 years), thus generating a population with an unparalleled genetic homogeneity. Up to date, this model has been used for research on several biological processes such as development (Seitz et al. 2005), neurobiology (Vilpoux et al. 2006), and epigenetics (Vogt et al. 2008). Furthermore, marbled crayfish has been shown to be very robust against various environmental parameters (Andriantsoa et al. 2019) and represents an excellent model system for modulating environmental conditions in a standardized laboratory setting.

## Future directions

Elucidating the role of epigenetic mechanisms in phenotypic plasticity and adaptation will remain an important research topic for many years to come. It will be important to avoid known issues in the design and interpretation of epigenome mapping studies that have plagued the field in the past (Lappalainen and Greally 2017; Lea et al. 2017). In this context, we consider three issues as particularly relevant: (1) Epigenetic patterns can be cell-type specific, but epigenetic profiles are often obtained from whole animals or bulk tissue. For example, two groups from different environments might differ in the cellular composition of a specific tissue, which could introduce a systematic (and potentially large) confounding effect that is unrelated to true epigenetic adaptation. As such, it is important to control the cellular sample composition. (2) Epigenetic effect sizes are often relatively small in ecological studies. The generation of conclusive results, therefore, requires the design of sufficiently powered studies with relatively high sequencing depths and relatively large sample numbers. We recommend that power estimates are included in the study design (Lea et al. 2017). (3) Wild specimens from a single species can have very heterogeneous genetic backgrounds, which can introduce a very strong confounding effect on the analysis. For example, if a cytosine is methylated in one population, but polymorphic in another population, it will be scored as differentially methylated. However, this change is not related to differential epigenetic programming, but rather reflects differences in population genetic structures. Integrated genome/epigenome analysis should be used to resolve this issue.

It will also be critically important to analyze the functional role of epigenetic mechanisms in rapid adaptation and invasiveness. This can be achieved by Clustered Regularly Interspaced Short Palindromic Repeats (CRISPR)-mediated editing (Rees and Liu 2018) and inactivation of various epigenetic modifier genes, such as DNA methyltransferases, DNA demethylases, and histone modifying enzymes. Knockout animals can then be challenged by changes in environmental parameters that can be easily varied in the laboratory (e.g., temperature, water supplements, and feed components). The increasing availability of complete genome sequences from non-model organisms and the broad application potential of CRISPR-based genome editing tools allow the use of functional approaches in an increasing number of interesting and relevant organisms.

Finally, it is also likely, that additional epigenetic mechanisms will emerge in the context of rapid adaptation and invasiveness. A prominent example is provided by small RNAs that can be loaded into oocytes and sperm and can thus be transmitted from parents to offspring (Chen et al. 2016). If these RNAs have the capacity to modulate genes that are involved in phenotypic plasticity, they can have a profound impact on the adaptive potential of the corresponding organism. Additionally, mRNA editing represents another interesting example of a mechanism that could conceivably play an important role in rapid adaptation (Eisenberg and Levanon 2018). A-to-I mRNA editing is a dynamic process that can diversify the transcriptome, and that has been strongly implicated in adaptive processes (Garrett and Rosenthal 2012). Interestingly, a tradeoff between genome evolution and transcriptome plasticity was suggested in cephalopods, as the genomic regions surrounding the editing sites appeared highly conserved (Liscovitch-Brauer et al. 2017). The high frequency and the conservation of editing sites significantly reduce genetic polymorphisms, and thereby decelerate genome evolution. Thus, RNA editing could provide a paradigm for how animals overcome the lack of genetic diversity and rapidly adapt to new environments by diversifying their transcriptome.

## Notes

From the symposium “SICB Wide Symposium: Building Bridges from Genome to Phenome: Molecules, Methods and Models” presented at the annual meeting of the Society for Integrative and Comparative Biology January 3–7, 2020 at Austin, Texas.
